# The effect of preoperative ultrasound localization on the incidence of infrapatellar branch of the saphenous nerve injury after hamstring tendon harvesting

**DOI:** 10.1186/s13018-025-05689-0

**Published:** 2025-03-26

**Authors:** Tianli Du, Jianfeng Chen, Chao Yan, Hongzhi Fang, Zhenghui Shang

**Affiliations:** The First College of Clinical Medical Science, China Three Gorges University, Department of orthopedics, Yichang Central People’s Hospital, Yichang, 443002 Hubei China

**Keywords:** Ultrasound guidance, Infrapatellar branch of the saphenous nerve, Sensory deficit, Anterior cruciate ligament reconstruction, Hamstring tendon

## Abstract

**Background:**

The potential of ultrasound-guided labelling of the inferior patellar branch of the saphenous nerve (IPBSN) to reduce IPBSN injury during anterior cruciate ligament reconstruction (ACLR) has not been explored. The primary objective of this retrospective cohort analysis was to assess whether intraoperative hamstring tendon harvesting avoiding the marked IPBSN would be effective in reducing the incidence of postoperative skin sensory disturbances and the mean area of sensory disturbances.

**Methods and analysis:**

A retrospective cohort study involving 60 patients who underwent autograft ACLR at Yichang Central People’s Hospital from October 2020 to October 2024 was conducted. Patients were divided into two groups on the basis of the use of preoperative ultrasound localization of the IPBSN, including the nonultrasound localization group (control group) and the ultrasound localization group (experimental group), with 30 patients in each group. The control group underwent standard ACLR with a diagonal incision for hamstring tendon harvesting, whereas the experimental group underwent preoperative ultrasound-guided localization of the IPBSN to avoid the nerve during incision. The primary outcome measures include the incidence of skin sensory disturbances and the average sensory disturbance area. The secondary outcomes include the Lysholm score and VAS score at the 6-month postoperative follow-up.

**Results:**

The incidence of skin sensory disturbances in the experimental group was lower than that in the control group, and the average area of sensory disturbance was smaller in the experimental group (*P* < 0.05). At the 6-month postoperative follow-up, no statistically significant differences in the Lysholm knee scores or visual analogue scale (VAS) pain scores were noted between the two groups (*P* > 0.05).

**Conclusion:**

Preoperative ultrasound-guided localization of the IPBSN can reduce the risk of nerve injury during ACLR. The ultrasound-guided approach leads to a lower incidence of sensory disturbances and a smaller average area of sensory disturbance. IPBSN injury was not related to anterior knee pain or knee ROM limitations. Patients can choose whether to use ultrasound localization before surgery according to their needs. The study protocol adhered to strict standards of ethical conduct and patient safety. The results of this trial are expected to provide valuable insights into the prevention of injury to the IPBSN during hamstring tendon harvesting.

## Background

Anterior cruciate ligament (ACL) injury is a common knee joint injury that typically presents with severe pain, swelling, and restricted movement in the acute phase; however, later stages can result in knee instability. Persistent instability during daily activities is an indication for ACLR, which aims to restore knee joint stability and prevent secondary meniscal and cartilage damage [1]. Arthroscopy-assisted ACLR is one of the most commonly performed orthopaedic procedures and is capable of restoring normal knee kinematics and ligament stability [2]. Currently, hamstring tendons are widely used in autograft tendon transplantation because of their ease of harvesting, making them one of the primary sources for grafts [3]. Compared with other methods, the use of hamstring tendons for grafts achieves similar clinical outcomes and is more convenient in terms of surgical technique. However, harvesting hamstring tendons can cause damage to the saphenous nerve, particularly the IPBSN, which may result in sensory deficits in the anterior tibial region and neuropathic pain [4]. The location of the hamstring tendon harvest site is closely related to the IPBSN, and the exact position and direction of the incision can influence the risk of IPBSN injury [5]. Therefore, special attention must be given to the protection of surrounding soft tissues when selecting the site for surgical incision and during the procedure to minimise the risk of IPBSN injury. This study analysed 60 patients who underwent ACLR and compared the outcomes on the basis of whether preoperative ultrasound-guided IPBSN localization was performed. It also assessed postoperative sensory deficit areas to investigate the relationship between ultrasound-guided ACLR and IPBSN injury.

## Methods

### Participants

This was a retrospective cohort study. A total of 60 patients who underwent autograft hamstring tendon reconstruction for ACL injury at Yichang Central People’s Hospital (Xiling District) between October 2020 and October 2024 were included. The patients were divided into two groups based on whether ultrasound-guided localization of the IPBSN was performed preoperatively, including the nonultrasound localization group (control) and the ultrasound localization group (experimental), with 30 patients in each group. In the control group, conventional arthroscopic ACL reconstruction was performed with a standard oblique incision for harvesting the hamstring tendon. In the experimental group, preoperative ultrasound guidance was used to localise the IPBSN. The direction of the IPBSN was marked on the skin with a pen. During ACL reconstruction, the surgical incision was made while avoiding the marked area. The incidence of sensory disturbances and the average area of sensory loss were compared between the two groups.

### Inclusion criteria

1. Patients with a confirmed diagnosis of ACL injury on the basis of clinical examination and imaging findings.
2. Patients who underwent ACL reconstruction using autograft hamstring tendons.
3. Surgeries performed by experienced surgeons at the associate chief physician level or higher.
4. Patients who were informed about the study and who voluntarily consented to participate.

### Exclusion criteria

1. Patients with pre-existing sensory or motor dysfunction in the lower limbs, such as lower limb deep vein thrombosis.
2. Patients with severe cardiovascular, cerebrovascular, or haematological disorders.
3. Patients with known allergies or systemic autoimmune diseases.
4. Patients with a history of prior knee surgery.

### Ultrasound-guided localisation

**Fig. 1:**
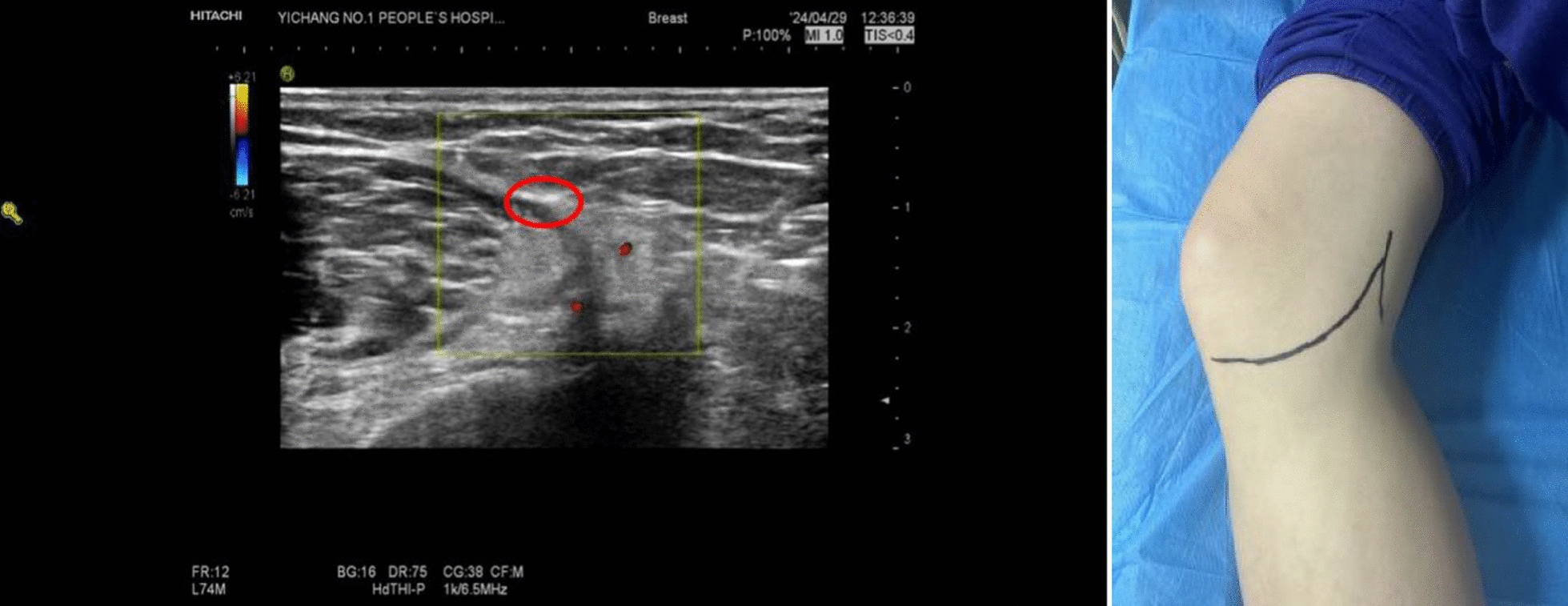
Preoperative ultrasound-guided localisation

The patient was placed in a supine position with the knee slightly flexed and supported by a cushion or pillow. An ultrasound probe was used to locate the anatomical structures in the knee area. The probe was positioned transversely 5 cm above the medial aspect of the femur, near the patella. The probe was slowly moved medially until the sartorius muscle, which is located in the midsection of the femoral medial muscle, appeared in view. The direction and angle of the probe were adjusted to clearly display the muscle structures of the sartorius, long adductor, and femoral medial muscles, with the pulsating femoral artery visible between them. A spindle or oval-shaped hyperechoic image of the saphenous nerve could be observed near the femoral artery. As the saphenous nerve lies on the lateral side of the femoral artery as it enters the adductor canal, it crosses over the artery to the medial side. When the probe is placed near the proximal end of the adductor canal, the nerve is typically found on the superior–lateral side of the femoral artery. However, if the probe is placed distally, the nerve is seen on the superior–medial side. Using the ultrasound image, the IPBSN and its terminal branches were identified, and the approximate path of the nerve was marked on the skin with a pen, as shown in Fig. 1.

### Surgical technique

Both groups underwent the same surgical procedure, utilizing the ipsilateral hamstring tendon as an autograft, which was prepared in a four-strand configuration. The femoral tunnel was created at the anatomical landmark with the knee flexed to 120°. The tibial tunnel was prepared with the knee flexed to 40° using an ACL tibial guide. A slanted incision was employed in both groups for access.

### Postoperative rehabilitation and bracing protocol

In the early postoperative phase, all patients were instructed to actively engage in knee joint mobilisation exercises. Within the first week after surgery, patients commenced quadriceps isometric contractions, straight leg raises, and ankle pumps. By the end of the first week, the range of motion (ROM) of the affected knee should reach 30° of flexion and extension, with a target of increasing the ROM by 15° per week until the optimal functional range is achieved. Between 2 and 6 weeks postoperatively, emphasis was placed on further improving joint mobility, managing symptoms, and initiating proprioceptive exercises. Gradual restoration of a normal gait was a key focus during this period. Patients were required to attend regular follow-up visits, during which the treating physician assessed knee symptoms and lower limb function to determine the appropriate time to return to sports activity. Preoperative education included detailed instructions on proper brace usage and care. The brace was applied immediately after surgery and worn as per the prescribed guidelines.

## Observational indicators

### Measurement of surgical incision

The length of the incision was measured using a tape measure.

### Measurement and analysis of skin sensory impairment areas

**Fig. 2:**
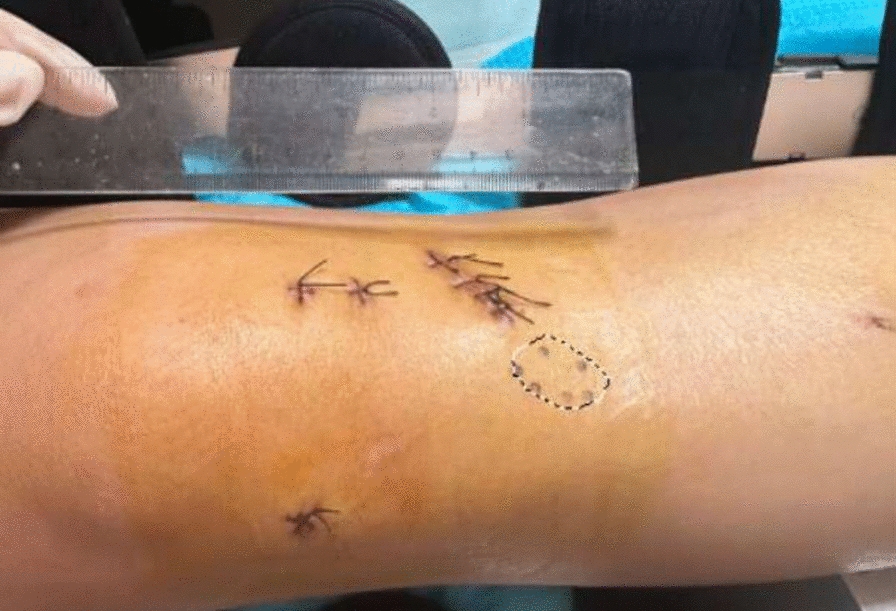
Measurement of sensory disturbance in the skin after surgery

The incidence rate and average area of sensory impairment were compared between the two groups. The method for measuring skin sensory impairment was as follows. The impaired sensory area on the patient’s skin was marked with a marker pen, as shown in Fig. 2. A photograph was then taken, and the area of sensory impairment was measured on a computer. Adobe Photoshop Elements 2.0 software was used to select the impaired sensory area, and the actual area of sensory impairment was calculated on the basis of the scale provided in the photograph.

### Postoperative knee joint function and patient satisfaction survey

Knee Joint Function Evaluation using the Lysholm score as follows: (1) pain, 25 points; (2) swelling, 10 points; (3) support, 5 points; (4) squatting, 5 points; (5) locking, 15 points; (6) stair climbing, 10 points; (7) limping, 5 points; and (8) joint instability, 25 points. The total score is 100 points. Scores are classified as follows: 90–100, excellent; 80–89, good; 70–79, fair; below 70, poor. Pain was evaluated via the visual analogue scale (VAS): (1) 0 points, no pain; (2) below 3 points, mild pain but tolerable; (3) 4–6 points, pain present and affects sleep, yet still tolerable; and (4) 7–10 points, gradually intensifying pain that is intolerable and affects appetite and sleep.

### Statistical analysis

Quantitative variables were analysed via the mean ± ST, whereas qualitative variables were reported as numbers and frequencies. An independent sample t test was used for comparisons between quantitative variables among the groups, whereas the chi square test (*χ*^2^) was used to compare the qualitative variables among the groups. *P* < 0.05 was considered statistically significant. All analyses were performed using SPSS 22.0.

## Results

### Comparison of the general conditions between the two groups of patients

There were no statistically significant differences between the two groups of patients in terms of sex, age, or body mass index (BMI). In the experimental group, the length of the oblique incision in 30 patients was 3.04 ± 0.25 cm, whereas it was 3.13 ± 0.31 cm in the control group. No statistically significant differences were noted between the two groups (*P* > 0.05), which eliminates confounding bias due to the length of the surgical incision causing skin sensory disturbances (Table 1).

**Table 1:** Comparison of baseline characteristics between the two groups of patients (*n* = 30)

Baseline data	Control group	Experimental group	Statistical value	*P* value
Gender (male/female, n)	18/12	15/15	*χ*^2^ = 0.61	0.436
Age ($\overline{{\text{x}}}$ ± s, years)	34.73 ± 14.08	32.03 ± 10.73	*t* = 0.87	0.386
BMI ($\overline{{\text{x}}}$ ± s, kg/m^2^)	25.24 ± 3.73	24.06 ± 3.62	*t* = 1.24	0.218
Incision length ($\overline{{\text{x}}}$ ± s, cm)	3.13 ± 0.31	3.04 ± 0.25	*t* = 1.22	0.227

### Comparison of the incidence rate and average area of skin sensory disturbances between two groups of patients

Among the 60 patients, 10 (11.7%) experienced skin sensory disturbances. In the control group, 8 patients (26.7%) experienced sensory disturbances, with an average area of 12.23 ± 2.51 cm^2^. In the experimental group, 2 patients (6.7%) experienced sensory disturbances, with an average area of 5.95 ± 1.28 cm^2^. Preoperative ultrasound-guided localisation of the IPBSN can prevent intraoperative injury (Table 2).

**Table 2:** Comparison of the incidence rates and average areas of skin sensory disturbances between the two groups (*n* = 30)

Indicator	Control group	Experimental group	Statistical value	*P* value
Incidence rate of skin sensory disturbances [n (%)]	26.7% (8/30)	6.7% (2/30)	*χ*^2^ = 4.32	0.038
Average area of sensory disturbances ($\overline{{\text{x}}}$ ± s, cm^2^)	12.23 ± 2.51 cm^2^	5.95 ± 1.28 cm^2^	*t* = 3.33	0.010

### Comparison of lysholm score and VAS at the 6-month postoperative follow-up between the two groups

At the 6-month postoperative follow-up, there were no statistically significant differences in the Lysholm knee scores or visual analogue scale (VAS) pain scores between the two groups (*P* > 0.05) (Table 3).

**Table 3:** Comparison of the incidence of skin sensory disturbances and the average area of sensory disturbances between the two groups

Indicator	Control group	Experimental group	Statistical value	*P* value
Lysholm score	86.43 ± 6.22	84.80 ± 5.33	*t* = 1.09	0.79
Visual analogue scale	0.57 ± 0.68	0.43 ± 0.63	*t *= 0.28	0.43

## Discussion

The infrapatellar branch of the saphenous nerve is a peripheral branch of the femoral nerve that originates from the L3–L4 spinal segments. The femoral nerve passes through the femoral triangle and gives rise to multiple branches, extending towards the medial side of the lower leg [6]. The IPBSN traverses the popliteal fossa, passes through the sartorius muscle, and then bifurcates downwards to supply sensation to the infrapatellar fat pad and the skin on the medial side of the knee [7]. Damage to the IPBSN can lead to pain, sensory disturbances, and numbness in the anterior medial knee area and can potentially affect knee joint movement [8]. The IPBSN runs deep within the knee joint and is enveloped by dense connective tissue, making it anatomically inconspicuous and vulnerable to iatrogenic injury from incisions and soft tissue dissection during both open and arthroscopic surgeries [9]. Therefore, for patients undergoing ACLR, it is crucial to have a detailed understanding of the anatomical position of the IPBSN preoperatively to preserve its integrity. This approach helps minimise postoperative complications and prevents inadvertent damage to the IPBSN.

Owing to iatrogenic factors being the main cause of injury to the IPBSN, measures to avoid such injuries have become a hot topic in current research [10–12]. During ACLR, the location and direction of the tendon incision are crucial to avoid IPBSN damage [13]. The IPBSN is closely associated with the proximal aspect of the gracilis tendon, and their travel directions are nearly parallel. During ACLR with the gracilis tendon as a graft under arthroscopy, a vertical incision increases the incidence of IPBSN injury due to its crossing path with the IPBSN. Conversely, an oblique incision reduces the incidence as it aligns with the direction of the IPBSN [14]. Keyhani [11] compared the incidence and area of skin sensory disturbances after vertical and oblique incisions and reported a lower incidence rate with oblique incisions, contrary to the findings of Leiter [15], who reported no significant difference in sensory disturbances between the two incision types. The rate of IPBSN injury depends on the alignment of the horizontal and oblique incisions with the IPBSN anatomy. Furthermore, major trunk injuries to the saphenous nerve may occur during the use of tendon extractors to harvest the gracilis tendon, potentially resulting in extensive sensory disturbances [16]. When the gracilis tendon is harvested, maintaining knee flexion and hip external rotation helps to avoid saphenous nerve injury caused by the tendon harvesting device, as the nerve shifts posteriorly and lies away from the incision site [17]. Therefore, precise preoperative planning of the incision site and meticulous surgical technique can greatly protect the integrity of the IPBSN. High-resolution ultrasound offers advanced real-time imaging capabilities, effectively displaying the trajectory of the IPBSN [18]. In normal nerve transections, ultrasound images reveal a circular hypoechoic bundle surrounded by a hyperechoic nerve sheath, creating a "honeycomb" appearance. Longitudinal views reveal multiple linear hypoechoic bundles arranged in parallel and separated by thin hyperechoic bands in the nerve sheath [19]. During exploration, when the saphenous nerve superficially exits the subcutaneous tissue, similar echogenicity between the nerve and surrounding tissues makes ultrasound visualisation challenging. Thus, the great saphenous vein is commonly used as an anatomical landmark to help locate the saphenous nerve or its branches around the vein. However, owing to the small size of the two terminal branches of the IPBSN and the potential presence of highly echogenic subcutaneous fat in the surrounding area, the use of ultrasound imaging to assess the terminal branches of the IPBSN presents significant challenges [20]. Therefore, under ultrasound guidance, efforts are made to explore the IPBSN and its terminal branches as thoroughly as possible, marking them on the patient’s skin surface with a marking pen and outlining the approximate course of the IPBSN to avoid it during surgery by making an oblique incision when the gracilis tendon is harvested. In this study, 8 cases of sensory disturbances occurred in the control group, and 2 cases were noted in the experimental group. All patients’ areas of sensory disturbance corresponded to the distribution area of the IPBSN, indicating that preoperative ultrasound-guided marking of the IPBSN can reduce the incidence of IPBSN injury. Some studies have followed up with IPBSN injury patients at 3, 6, 12, and 24 months postoperatively, observing changes in the area of sensory disturbance. The results revealed a decreasing trend over time, regardless of whether vertical or oblique incisions were used [15]. This finding is consistent with the results of Zhu et al. [17], although the sensory disturbance did not fully resolve during the follow-up period. This could be due to excessive stretching of the incision to expose the tendon, leading to blunt nerve traction injury. Studies have shown that postoperative sensory recovery is related to avoiding excessive stretching of the incision and carefully closing the wound [17]. In the present study, the follow-up at 6 months postsurgery in the control group revealed no reduction in the area of sensory disturbance. However, in the experimental group, one patient showed a reduction in the area of sensory disturbance at 6 weeks postsurgery with pain sensation restored, although superficial sensation had not yet recovered. By 6 months postsurgery, the area of sensory disturbance in this patient had almost disappeared. This situation might be due to traction injury of the IPBSN caused by incision tension during surgery. There are two possible reasons for intraoperative injury associated with preoperative ultrasound-guided localisation of the IPBSN. First, although preoperative ultrasound localisation can clearly visualise the main trunk of the nerve, it is less effective for detecting smaller branches less than 1 mm in diameter. Thus, the nerve can be identified only by its anatomical relationship with surrounding tissues. Second, during hamstring tendon harvest, the patient’s position may differ from that during preoperative ultrasound scanning, which could result in slight displacement of the nerve. In this study, the position of the IPBSN marked on the skin before surgery was slightly affected by the patient’s change in position, but the displacement typically did not exceed 1 mm. In our study, the differences in the visual analogue scale and Lysholm scores between the two groups at 6 months after surgery were not statistically significant (*P* > 0.05). We found that not all patients with infrapatellar branch saphenous nerve injury experience tingling, and only a minority of patients experience this symptom, which is affected by the injury mechanism, individual nerve sensitivity, and repair capacity. In contrast, skin numbness is more common but does not affect the function of the knee joint.

## Limitations

Although preoperative ultrasound localisation can clearly visualise the main trunk of the nerve, it is less effective for detecting smaller branches less than 1 mm in diameter. Thus, the nerve can be identified only by its anatomical relationship with surrounding tissues. During hamstring tendon harvest, the patient’s position may differ from that during preoperative ultrasound scanning, which could result in slight displacement of the nerve. In this study, the position of the IPBSN marked on the skin before surgery was slightly affected by the patient’s change in position, but the displacement typically did not exceed 1 mm. The cost is also a constraint. In China, the cost of ultrasound examination is relatively economical, and it is possible to communicate with patients and decide whether to use ultrasound before surgery according to their needs to reduce the risk of saphenous nerve infrapatellar branch injury.

## Conclusion

Preoperative ultrasound-guided IPBSN labelling can effectively reduce the risk of nerve injury and the area and incidence of skin sensory disturbance, which has important clinical application value. Therefore, ultrasound-guided IPBSN marking can be considered an optional treatment strategy to guide surgical planning and deserves widespread clinical adoption.

## Data Availability

No datasets were generated or analysed during the current study.
